# The effect of foliar application of *Ascophyllum nodosum* (L.) Le Jol. seaweed extract on biochemical traits related to abiotic stresses in pistachio (*Pistacia vera* L. cv. Kaleh-Ghoochi)

**DOI:** 10.1186/s12870-023-04654-5

**Published:** 2023-12-11

**Authors:** Mohammadali Nikoogoftar-Sedghi, Vali Rabiei, Farhang Razavi, Sanaz Molaei, Ali Khadivi

**Affiliations:** 1 Department of Horticulture, Faculty of Agriculture, University of Zanjan, Zanjan, Iran; 2 Department of Horticultural Sciences, Faculty of Agriculture and Natural Resources, Arak University, 38156-8-8349 Arak, Iran

**Keywords:** Pistachio, Seaweed, *Ascophyllum nodosum*, Tension, Enzymes

## Abstract

**Background:**

Due to the important economic role of pistachio (*Pistacia vera* L.) the cultivation of this valuable crop has been extended. Various abiotic stresses harm the growth and performance of pistachio. Seaweed extract containing various substances such as pseudo-hormones that stimulate growth, nutritional elements, and anti-stress substances can cause more resistance to abiotic stresses, and increase the quantity and the quality of the fruit. The present study was conducted to evaluate the effect of foliar application of *Ascophyllum nodosum* (L.) Le Jol. seaweed extract on some biochemical traits related to abiotic stress in *Pistacia vera* L. cv. Kaleh-Ghoochi. The first factor of foliar spraying treatment included *A. nodosum* seaweed extract at four levels (0, 1, 2, and 3 g/L), and the second factor was the time of spraying solution which was done at three times (1- at the beginning of pistachio kernel growth period at the end of June, 2- at the stage of full kernel development at the end of August, and 3- Spraying in both late June and August).

**Results:**

The results showed that all investigated traits were significant under the treatment of seaweed extract compared with the control. The seaweed extract concentrations had a significant effect on all traits except soluble carbohydrates, but the time of consumption of seaweed extract on soluble carbohydrates, protein, peroxidase, ascorbate peroxidase, and superoxide dismutase enzymes was significant, while had no significant effect on the rest of the traits. According to the interaction effect of time and concentration of consumption of seaweed extract, the highest values of the biochemical characters were as follows: total phenol content: 168.30 mg CAE/g DW, flavonoid content: mg CE/g DW, catalase: 12.66 µmol APX min^− 1^ mg^− 1^ protein, superoxide dismutase: 231.4 µmol APX min^− 1^ mg^− 1^ protein, and ascorbate peroxidase: 39.53 µmol APX min^− 1^ mg^− 1^ protein.

**Conclusions:**

Based on the results of this study, it seems that it is possible to use fertilizers containing *A. nodosum* seaweed extract with a concentration of 3 g/L in August to increase the tolerance of the pistachio cultivar “Kaleh-Ghoochi” to abiotic stresses.

## Introduction

Pistachio (*Pistacia vera* L.) is one of the important plants of the Anacardiaceae family and one of the most important nuts in the world. It is considered one of the important non-oil export crops in Iran. Due to the important economic role of pistachio in export and its key position in improving the economic situation, management and planning to increase the area of ​​cultivation, control abiotic stresses and increase the quantitative and qualitative yield of this crop seems necessary. According to the latest statistics of the World Food Organization (FAO), Iran has the largest cultivated area of ​​this crop in the world, but in recent years, the yield and quality of pistachios have gone down and its production has not kept pace with global growth, which improper nutrition of pistachio orchards [1] and abiotic stresses [2] are the major reasons of this situation.

In recent years, Iran, along with the USA and Turkey, had the highest amount of pistachio production, so in total they accounted for more than 83% of the world’s production [3]. Adaptability to all types of soils and climates has led to the development of the cultivation of this valuable crop in more than 22 provinces of Iran [4]. This planting development has caused pistachio trees to face various abiotic stresses, which harms their growth and performance. Plants are immobile organisms and therefore are affected by changing environmental conditions such as drought, storms, high temperatures, high salt concentration, heavy metals, high-intensity radiation, and pathogenic agents [5]. Statistics have shown that the area of ​​the world’s lands under drought stress has doubled from 1970 to 2000 [6] and the average decreased crop yield, caused by abiotic stresses in different parts of the world, is more than 50% [7]. Plants have many morphological and physiological mechanisms that adapt them to stress [8]. The defense system or antioxidant activity of the plant includes the activity of enzymes and non-enzymatic antioxidant compounds. Enzyme activity includes superoxide dismutase, catalase, ascorbate peroxidase, and glutathione reductase enzymes [9]. Non-enzymatic antioxidant compounds include vitamins, phenols, flavonoids, and anthocyanins [10]. Superoxide dismutase is the first enzyme involved in destroying free radicals, which causes the destruction of superoxide radicals to oxygen and hydrogen peroxide radicals, then catalase, ascorbate peroxidase, and glutathione reductase catalyze hydrogen peroxide to oxygen and water molecules, hence the increase the activity of superoxide dismutase enzyme can increase the ability of fruits to destroy reactive oxygen species [11]. The production of reactive oxygen species, changes in nitrogen and carbon metabolism, instability and destruction of cell membranes, metabolic toxicity, inhibition of photosynthesis, and reduction of nutrient absorption are some of the factors that lead to adverse events in plants [12]. Hydrogen peroxide is one of the types of active oxygen that is produced in plant cells in addition to stress conditions under normal conditions, and because it is neutral in terms of electric charge, it is easily transferred from one cell to another which can cause cell tissue destruction [13]. Reactive oxygen species are a natural product of plant metabolism that are created in cells in a natural state and play a role in cellular signaling with low concentrations, but when their amount increases under stress, they cause oxidative damage to cells. In non-stress conditions, there is a balance between the production of reactive oxygen species and the capacity to destroy these compounds by the plant’s defense system, but in stressful conditions, the production of reactive oxygen species is greater than the plant’s defense capacity to destroy them and as a result, oxidative stress occurs as secondary stress [14]. Various studies have shown that there is a strong relationship between tolerance to oxidative stress caused by abiotic stresses and there is an increase in the concentration of antioxidant enzymes in photosynthetic plants [15–17].

Seaweed extracts are widely used worldwide as one of the plant growth enhancers and due to having a large number of compounds that stimulate plant growth, they are used in the production of crops [18]. They contain various substances, including growth-stimulating pseudo-hormones, nutritional elements, and a variety of anti-inflammatory substances that affect the better development of roots, increasing the absorption of water and mineral salts, increasing the amount of chlorophyll, increasing flowering and fruit formation, creating more resistance against abiotic stresses, and increasing the quantity and quality of fruit [19]. Also, these compounds are mostly used to improve the growth and development of agricultural and horticultural plants and reduce the effect of abiotic stresses on them, and do not cause dangerous contamination for humans, animals, and birds [20]. *Ascophyllum nodosum* (L.) Le Jol. seaweed extract is well-known and commercially used in agriculture due to its ability to be used in organic agriculture and food production [21], and it can improve plant growth, yield, resistance to increased stress, and fruit quality [22]. This seaweed is one of the large and main types of marine seaweed [19], which can be a suitable alternative to chemical fertilizers to reduce environmental pollution and health problems caused by the consumption of chemical fertilizers [23]. Also, seaweed extract increases the tolerance of plants to a wide range of abiotic stresses such as salinity, drought, and high temperature. It has been reported that some seaweeds have plant growth-stimulating activity, and as a result, they are used as organic fertilizers in agriculture [24].

The use of seaweed extract increases plant growth, and the number of leaves, stimulates root growth, accelerates flowering time, increases fruit formation, delays leaf senescence, improves resistance to abiotic stresses such as drought, salinity, and temperature, and increases fruit quantity and quality [25, 26]. By stimulating growth in the early stages of plant growth, *A. nodosum* seaweed helps to develop and improve the growth of organs in the later stages of growth and increases the yield of the crops. An increase in the quantity and quality of fruit in response to this fertilizer in other crops has also been observed [27] Spraying seaweed is used both to increase plant growth and to withstand abiotic stresses such as frost, drought, and salinity, and to improve photosynthetic activity and plant performance [28].

Considering the indiscriminate use of chemical fertilizers and the adverse effects of these fertilizers on the environment, paying attention to the role of organic fertilizers and biological stimulants in food security and sustainable agriculture has become more important. In recent years, because of the decrease in yield of most fruit crops, including pistachio due to biotic and abiotic stresses, the use of supplementary fertilizers based on amino acids and seaweed has been increased greatly. Considering that abiotic stresses has always been one of the most important issues and problems of agriculture, it seems that the use of *A. nodosum* seaweed extract can partially reduce the negative effects of abiotic stresses in pistachio and improve its quantitative and qualitative performance. Thus, the present research was carried out to investigate the effect of foliar application of *A. nodosum* seaweed on some biochemical characters related to stress and enzyme activities of the pistachio cultivar “Kaleh-Ghoochi”.

## Materials and methods

### Plant material and area studied

The present research was conducted in the year 2021 in an orchard of Masoumeh complex in Jafariyah city, Qom province, Iran. Jafariyah has a semi-arid climate, an average annual rainfall of 136 mm, and an average annual temperature of 19 ˚C. The tested pistachio cultivar was “Kaleh-Ghoochi” grafted on Badami-Riz rootstock and the age of the trees was 20 years. The “Kalah-Ghoochi” is one of the most popular pistachio cultivars available in Iran. Due to the large size, this cultivar has attracted the attention of buyers in Iran and other countries more than other cultivars. In addition to its significant economic, nutritional, and medicinal values, this cultivar is compatible with abiotic stresses and as a plant that tolerates drought and salinity, it is a suitable crop for afforestation in dry and salty areas. Healthy trees with uniform growth were selected and the treatments were applied in the form of foliar spraying in the early morning with a 20-liter sprayer so that all the leaves of the trees were uniformly wet. The nutrition, irrigation, and other operations were carried out according to the calendar of previous years. Irrigation of the orchards was done with drip every 10 days.

### Treatments details

The research was done in factorial form in a randomized complete block design in three replications (three trees in each replication). The first factor included foliar spraying of *A. nodosum* seaweed extract at four levels of 0 (spraying solution with distilled water), 1, 2, and 3 g/L and the second factor was the time of spraying the solution which was done three times (1- at the beginning of the period pistachio kernel growth in late June 2- in the stage of full kernel development in late August and 3- spraying in both late June and August). Fruits were randomly sampled from different directions of the tree at the stage of physiological ripening.

The *A. nodosum* seaweed extract (produced by Acadian plant company made in Canada) in powder form and easily soluble in water, contains 45% organic matter, 17% potassium soluble in water, and a small amount of nitrogen and phosphorus. The fruits were harvested at the end of September and the stage of physiological ripening and were transferred to the post-harvest physiology laboratory of Horticultural Department, University of Zanjan, Zanjan province, Iran.

### The characteristics evaluated

Protein, carbohydrate, total phenol content, flavonoid content, antioxidant activity, catalase (CAT), ascorbate peroxidase (APX), superoxide dismutase (SOD), and peroxidase (POD) were evaluated according to conventional methods.

#### The soluble protein content

One g of the sample was placed into a Chinese mortar, then 25.60 ml of extraction buffer (Tris-HCl, pH = 7.5) was added to the sample in several steps and ground for 30 min. Then the sample was poured into a 15 ml Falcon. To better extract the protein and completely dissolve the sample in the buffer, the falcon tubes of the sample were kept stationary in the refrigerator for 24 h. Then the samples were centrifuged at 6000 rpm for 20 min. Then, 0.10 ml of the floating liquid phase of the samples was removed and poured into a 15 ml falcon, and five ml of Bradford’s reagent was added to it and vortexed rapidly. After 25 min, the optical absorbance of the resulting solution was read at 595 nm with a spectrophotometer (Model Specorp 250 Jena-History, Germany). Protein was calculated using a standard curve [29].

#### Soluble carbohydrates

Pistachio kernel (500 mg) was crushed in a Chinese mortar with 5 cc of 95% ethanol, and then the upper part of the solution was separated and the remaining sediments were re-extracted with 5 ml of 70% ethanol. The extract was centrifuged at 4500 rpm for 15 min. 100 µl of the extract was taken and 3 ml of anthrone (150 mg of pure anthrone + 100 ml of 72% sulfuric acid) was added to it. It was placed in a boiling water bath for 10 min and after cooling, the absorbance was read at 625 nm. Pure glucose with different concentrations was used as a standard and finally, the absorbance at 625 nm was read using a spectrophotometer [30].

#### Total phenol and flavonoid contents

The 1.00 g of pistachio kernel was weighed and pounded in a mortar by adding 80% methanol, brought to a final volume of 8 ml, and the pounded tissues were centrifuged for 10 min at 6000 rpm, and for measurement of flavonoid and total phenol contents.

Total flavonoid content of the extracts was evaluated using colorimetric method. After centrifugation, 75 µl of sodium nitrite (5% W/V), 0.15 ml of aluminum chloride (10% W/V) and 0.50 ml of sodium hydroxide were added to 0.25 ml of the extract, and by adding distilled water, the final volume was brought to 2.50 ml. The absorbance of the solution was read after 5 min at 507 nm a spectrophotometer (model Specorp 250 Jena-History) made in Germany [31]. The curve was drawn based on the amount of absorption in specific concentrations.

To measure total phenol content, after centrifugation, 0.10 ml of the extract was taken and 2 ml of 7% sodium carbonate was added to it. After 5 min, 0.10 ml of Folin’s reagent diluted (50%) was mixed. To calculate the standard curve, gallic acid was prepared in different concentrations and the absorption rate was recorded. Total phenol content was reported based on mg/g dry weight. The absorbance at 720 nm was read using a spectrophotometer [32].

#### Antioxidant activity

The 1.00 g of pistachio kernel was centrifuged in 8 ml of 80% methanol and then 50 µl of this extract was added to 1950 µl of solution (0.10 mmol) so that the final volume was two ml. To prepare 0.10 mM DPPH, 3.934 mg of DPPH was dissolved in 100 ml of methanol. Its absorbance was read after 10 min at 517 nm using a spectrophotometer. DPPH solution was used to compare the absorption of samples. DPPH solution without extract remains unchanged during this period, but the color of DPPH solution containing plant extract decreases over time and its absorption amount decreases compared with DPPH solution. The greater the antioxidant activity of the extracts, the greater the color reduction. The radical scavenging activity was calculated based on the percentage of DPPH radical scavenged (RSA) using the following equation [33].

% Total antioxidant = (OD control-OD sample)/OD control × 100.

OD sample: absorption of the sample containing the extract, OD control: absorption of the control

#### Enzyme activity measurement

For extract preparation for enzyme measurement, 1.00 g of frozen tissue was ground in a porcelain mortar containing three ml of 50 mM phosphate buffer (pH = 7) containing 1.00% polyvinyl pyrrolidine PVP and 1.00 mM EDTA. The resulting extract was placed in a refrigerated centrifuge at a temperature of 4 ˚C at 12,000 rpm for 20 min. A supernatant solution was used to study the activity of enzymes in the desired samples.

#### Catalase

Catalase enzyme activity was measured at 240 nm. The reaction mixture with a volume of 3 cc included 2.870 cc of potassium phosphate buffer and 30 µl of hydrogen peroxide, after which 100 µl of enzyme extract was added to the mixture. Absorption changes were calculated in 30 s from the start of the reaction to one minute after the start of the reaction. The activity of this enzyme was calculated based on the decreasing changes in hydrogen peroxide concentration and its breakdown in terms of one unit per mg of protein [34].

#### Ascorbate peroxidase

The activity of this enzyme was measured according to the method of Nakano and Asada [35]. The reaction mixture included 0.10 ml of enzyme extract, 0.20 ml of 3% hydrogen peroxide, 2 ml of 50 mM potassium phosphate buffer (pH = 7), and 0.20 ml of 0.05 mM ascorbate. With the start of the enzymatic reaction, the decrease in absorbance at 290 nm was calculated one minute after the start of the reaction and in 30-second intervals. Using the absorbance changes per minute, the extinction coefficient of ascorbate ε: 2.8 mM cm^− 1^ was calculated. One enzyme unit is the amount of enzyme that oxidizes one mM ascorbate in one minute. Enzyme activity was expressed as one unit per mg of protein.

#### Peroxidase

Peroxidase enzyme activity was measured using guaiacol. The reaction mixture consisted of 30 µl of 1% guaiacol, 300 µl of 3% hydrogen peroxide, and 2.65 cc of 50 mM potassium phosphate buffer (pH = 7). The reaction was started by adding 20 µl of enzyme extract to the reaction mixture and the absorbance was read at 470 nm in 30-second intervals and after one minute. Extinction coefficient ε: 25.5 mM cm-1. Enzyme activity was expressed as one unit per milligram of protein [36].

#### Superoxide dismutase

The activity of superoxide dismutase enzyme was performed using the method of Giannopolitis and Ries [37]. The reaction mixture consisted of 25 mM sodium phosphate buffer (pH = 6.8), 13 mmol of methionine, 0.1 mmol of EDTA, 0.075 µmol of nitrobuterazolium, 75 micromol of riboflavin and 50 µl of enzyme extract. After exposing the reaction mixture to fluorescent light for 20 min (40 W for 8 min at 25 degrees), it was read at 560 nm. The test tube without enzyme extract was used as a control or as a comparison reference with the test tube (with enzyme extract with similar light intensity). The reaction mixture without light treatment was used to zero the device. The activity of one unit of superoxide dismutase enzyme was considered as the amount of enzyme that leads to 50% inhibition in the photoreduction of nitrobuterazolium and was expressed as one unit per mg of protein.

### Statistical analysis

Analysis of variance (ANOVA) and comparing the averages using Duncan’s multi-range test method at the 0.05 probability level were performed with SAS (Version 6) software [38]. Drawing graphs and tables was done using Excel software. In figures, columns with a common letter of the mean comparison test have no statistically significant difference at the 0.05 probability level. Also, the vertical bars represent the standard errors of the means.

## Results and discussion

The results obtained from the analysis of variance (Table 1) showed that the application of seaweed extract had a significant effect on soluble carbohydrates, soluble protein content, total phenol content, flavonoid content, antioxidant activity, catalase, superoxide dismutase, ascorbate peroxidase, and peroxidase, while consumption time of seaweed extract had a significant effect only on soluble carbohydrate, soluble protein, superoxide dismutase, ascorbate peroxidase, and peroxidase. The interactions of these treatments were also significant on total phenol, flavonoid, catalase, superoxide dismutase, and ascorbate peroxidase.

**Table 1:** The results of analysis of variance of the effect of *Ascophyllum nodosum* seaweed extract treatments on biochemical traits in pistachio “Kaleh-Ghoochi”

						Mean squares				
Source	Df	Soluble Protein	Soluble carbohydrates	Antioxidant activity	Total flavonoid	Total phenols	Peroxidase	Catalase	Ascorbate peroxidase	Superoxide dismutase
**Block**	2	5.13 ^ns^	75.53*	79.73 ^ns^	63.75*	243.7 ^ns^	0.03 ^ns^	6.42*	21.34 ^ns^	176.85 ^ns^
**Time**	2	19.86*	63.88*	51.13 ^ns^	14.40 ^ns^	119.3 ^ns^	0.13*	4.7 ^ns^	39.77**	6049.50*
**Time × Block**	4	0.79 ^ns^	14.47 ^ns^	22.20 ^ns^	18.56 ^ns^	43.9 ^ns^	0.03 ^ns^	6.05*	7.38 ^ns^	1164.7 ^ns^
**Concentration**	3	11.21*	118.51 ^ns^	670.40 **	379.4**	1353.90**	0.82**	40.44**	256**	33774.80**
**Interaction effect**	6	6.72 ^ns^	31.01 ^ns^	100.5 ^ns^	107.3**	669.30**	0.08 ^ns^	13.33**	123.45**	3255.30*
**Concentration × Block**	6	0.41 ^ns^	3.28 ^ns^	64.12 ^ns^	80.73**	348.10*	0.02 ^ns^	1.40 ^ns^	22.82 ^ns^	399.9 ^ns^
**Error**	12	2.96	13.38	57.97	13.99	103.9	0.33	1.27	14.27	882.53
**CV (%)**	-	15.51	15.44	10.73	3.71	7.24	22.45	12.56	13.74	18.96

ns, *, and **: non-significant, significant at *P* ≤ 0.05, and significant at *P* ≤ 0.01, respectively

### Soluble protein content

**Fig. 1:**
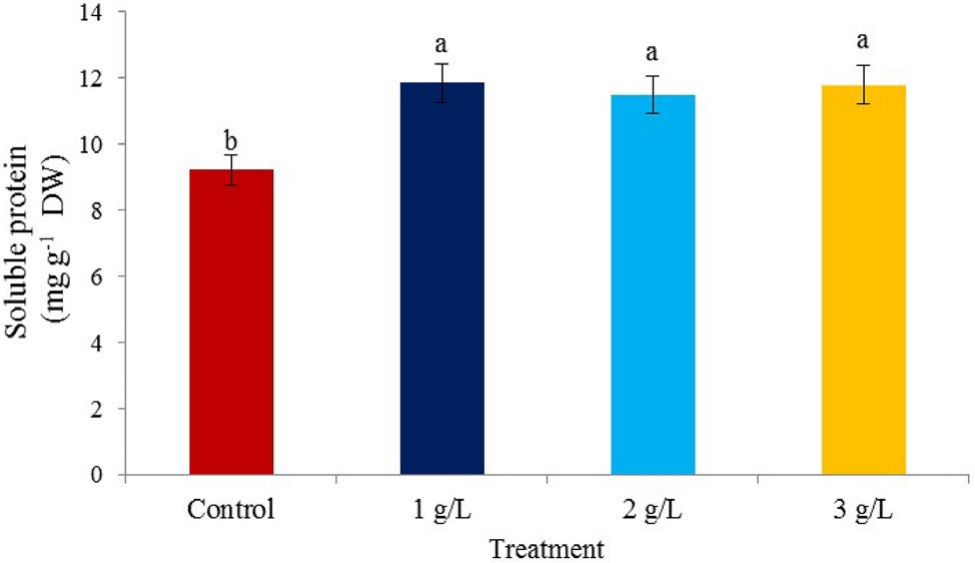
The effect of different *Ascophyllum nodosum* seaweed extract concentrations on soluble protein in pistachio “Kaleh-Ghoochi”

There is a significant difference in the protein content between the time treatments and the consumption of *A. nodosum* seaweed with the control. The concentration of seaweed extract showed a significant difference on protein content in all three treatments compared with the control (Fig. 1). The highest content of protein was 11.85 mg/g DW of pistachio in the treatment of 1 g/L of seaweed extract and the lowest was observed in the control at 9.22 mg/g DW of pistachio. The increase in protein in pistachios, which was observed due to the consumption of concentrations (1, 2, and 3 g/L) of seaweed extract, showed the effect of seaweed extract in increasing tolerance to abiotic stresses. Dionne et al. [39] reported that protein can regulate the expression of genes responsible for stress tolerance, and under conditions of low temperatures, cultivars tolerant to freezing stress accumulate more soluble protein than cultivars with less tolerance. *A. nodosum* seaweed extract treatment increased the soluble proteins in spinach [40]. Also, seaweed extract treatment increased protein production [41] which is consistent with the results of the present study. The application of seaweed extract in onions increased the plant height, the number and size of onions, the percentage of sulfur and protein in onions, and the concentration of chlorophyll and carotenoids in leaves [42].

**Fig. 2:**
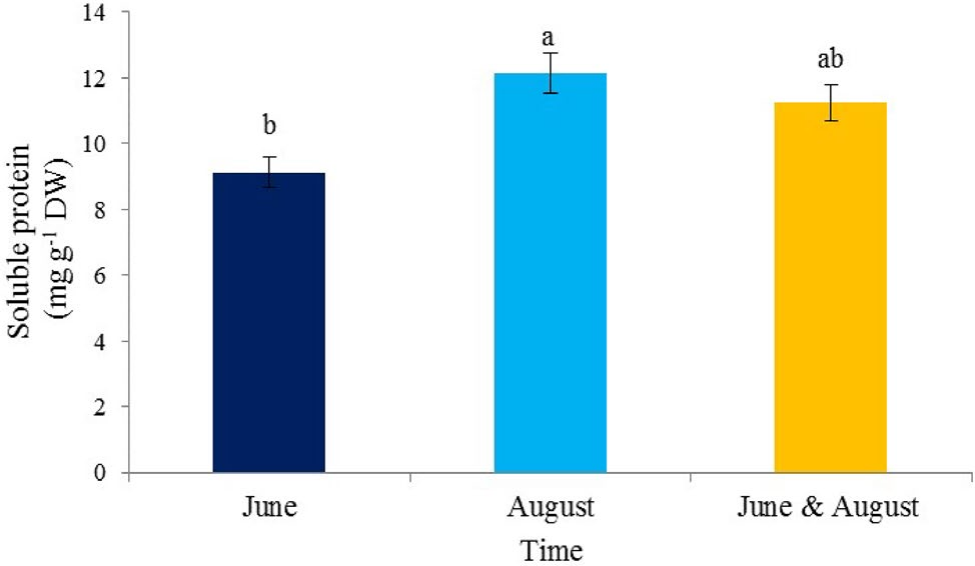
The effect of the time of application of *Ascophyllum nodosum* seaweed extract on soluble protein in pistachio “Kaleh-Ghoochi”

A significant difference was observed between the treatments in the times of consumption of seaweed extract (Fig. 2). The highest protein content was observed in August (12.13 mg/g DW) and the lowest in June (9.12 mg/g DW).

Amino acid production is possible by spending a lot of energy in the plant, and the plant does not spend its energy in making amino acids in times of stress and obtains its amino acids by breaking proteins, because this process requires less energy than amino acid production. Therefore, the use of seaweed before, during, and after the occurrence of abiotic stresses such as heat, cold, and drought, and excessive use of toxins can save the plant’s energy for survival [43]. It seems that increasing protein content in the seaweed extract treatment in August is done to protect the proteins and prevent their decomposition. Also, seaweed extract affects the activity of enzymes effective in the absorption and use of nitrogen in the plant and leads to an increase in the protein content in the plant [44, 45]. The consumption of nitrogen fertilizers in August, which was used for pistachios, can lead to an increase in protein in August. According to previous reports, the increase in the protein content maybe due to the presence of acetic acid or similar compounds and also due to the presence of growth stimulants in seaweed extract [46].

### Soluble carbohydrates

**Fig. 3:**
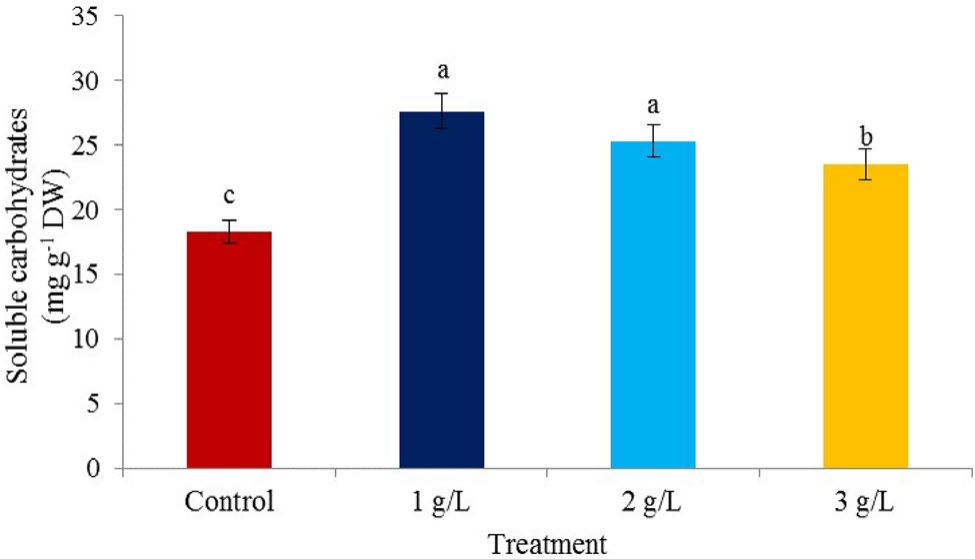
The effect of different *Ascophyllum nodosum* seaweed extract concentrations on soluble carbohydrates in pistachio “Kaleh-Ghoochi”

The treatment of one g/L seaweed extract had the greatest effect on the soluble carbohydrates of the kernel (62.27 mg/g of DW) and the lowest content in the control treatment (28.18 mg/g DW) was observed (Fig. 3). The accumulation of carbohydrates can play an important role in protecting plant cells from damage caused by stresses in fruit trees [47]. During active photosynthesis in the presence of light, carbohydrate content produced in the form of triosephosphate in the leaf is more than the content required for energy production and compound synthesis. Excess carbohydrates are converted into sucrose and transferred to other parts of the plant [48]. The positive effect of seaweed is probably due to the presence of growth-stimulating hormones, minerals, and nutrients that increase the chlorophyll content in the leaves and the photosynthetic activity of the plant, and as a result, it causes an increase in carbohydrates in pistachio. The use of the extract of this seaweed increases the concentration of total chlorophyll in plant leaves and increases the level of amylase enzyme in plant organs, thus breaking down unusable sugars in the plant [49].

### Total phenol content

**Fig. 4:**
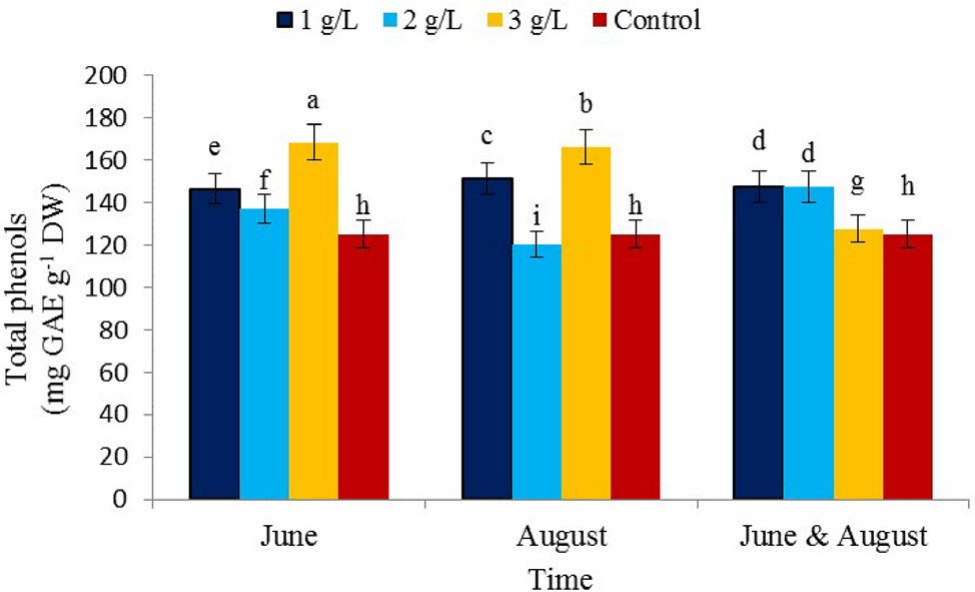
The effect of the time of application of *Ascophyllum nodosum* seaweed extract on total phenols in pistachio “Kaleh-Ghoochi”

The interaction effects of treatment of 3 g/L of seaweed extract in June showed the highest content (168.3 mg CAE/g DW) and treatment of 2 g/L in August showed the lowest content of total phenol (3 120 mg CAE/g DW) (Fig. 4). Secondary plant metabolites can protect the plant against stressful conditions and also improve human health and nutrition [50]. Phenols are the largest group of secondary metabolites that are produced from pentose phosphate and shikimic acid and phenylpropanoid pathway in plants [51]. The mechanism of action of plant phenolic compounds that act as antioxidants mainly includes free radical scavenging activity, chelating properties, ability to regulate gene expression, and antioxidant role [52]. The results of the study of Frioni et al. [35] regarding the foliar spraying of seaweed extract with a concentration of 1.5 kg per hectare on Pinot-Noir grapes in the Mediterranean climate in central Italy and Cabernet Franc in the cold climate of the state of Michigan, USA, showed that the application of the extract seaweed increased the phenolic compound content in grapes. The use of *A. nodosum* seaweed extract treatment increased the total phenol content in spinach [53] and *Triticum aestivum* var. Pusa Gold [54] under oxidative stress conditions. Seaweeds, especially brown seaweed, are rich in phenolic compounds [55] which is consistent with the present study.

### Total flavonoid content

**Fig. 5:**
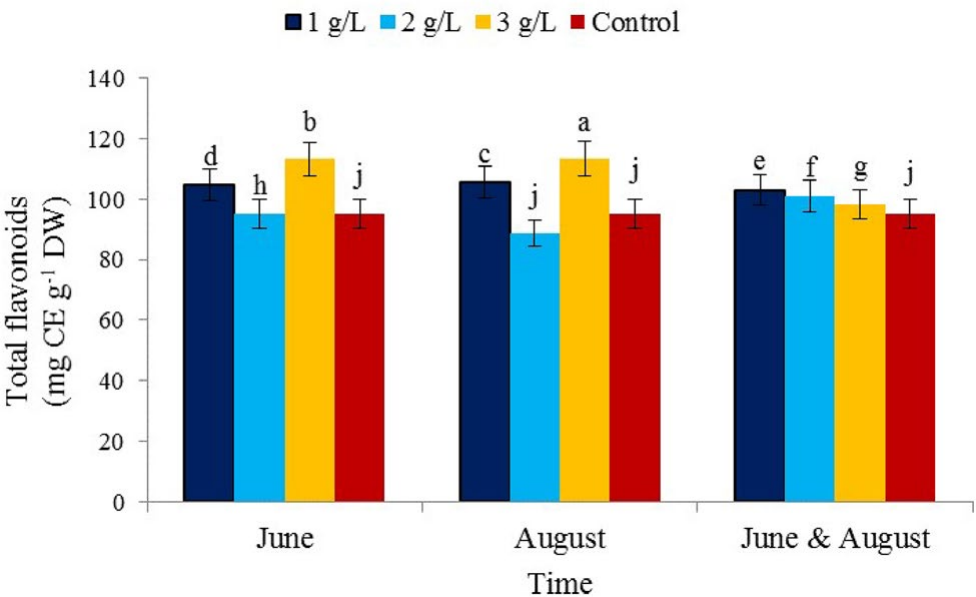
The effect of the time of application of *Ascophyllum nodosum* seaweed extract on total flavonoid content in pistachio “Kaleh-Ghoochi”

The interaction effects of the treatment of 3 g/L of seaweed extract in August) showed the highest content (113.30 mg CE/g DW) and the concentration treatment of 2 g/L in August showed the lowest content (88.80 mg CE/g DW) of flavonoid (Fig. 5). One of the main reasons is the importance of flavonoids in their function in the defense system. Environmental factors have a significant effect on the activity of flavonoids. It seems that potassium increases flavonoids due to its high physiological activity in plant structure and reducing water consumption [56]. The results of other investigations have reported the presence of phenolic compounds (flavonoids and anthocyanins) in pistachio kernels and the antioxidant properties of these compounds [57, 58]. Flavonoids strengthen the effect of ascorbate and protect it from other substances that are easily oxidized. Flavonoids have a polyphenol structure with the ability to remove free radicals. Due to their hydrophilic nature, these compounds are located in vacuoles and partly in the cell wall, that is, where reactive oxygen groups (ROS) penetrate less.

### Antioxidant activity

**Fig. 6:**
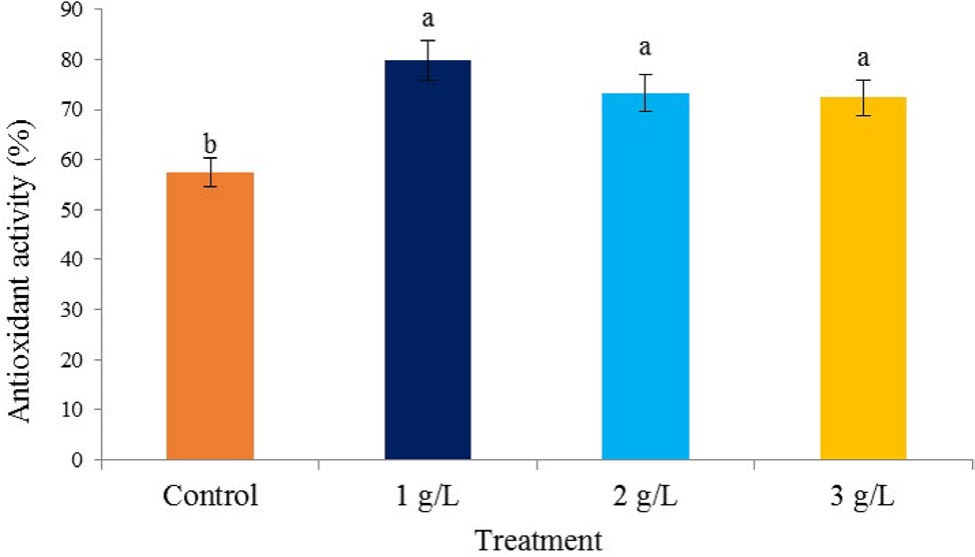
The effect of different *Ascophyllum nodosum* seaweed extract concentrations on antioxidant activity in pistachio “Kaleh-Ghoochi”

The highest percentage of inhibition of free radicals was observed in the treatments of 1, 2, and 3 g/L of seaweed extract (79.87, 73.35, and 72.72%, respectively), and the lowest was observed in the control (57.34%) (Fig. 6). Antioxidant activity is important in plants that plays an important role in preventing the transformation of hydroxides into free radicals [59]). Depending on their genetic capacity, plants expand their antioxidant defense system to deal with reactive oxygen species [60]. One of the most important goals of enzymatic and non-enzymatic antioxidant systems is to neutralize reactive oxygen species during stress [61]. Marine seaweeds, especially brown seaweed, are rich in thymolic compounds [62]. Thymolics are secondary metabolites synthesized under stress conditions that protect cellular components. The important role of thymolic compounds includes antioxidant activity, the inhibition of free radicals such as singlet oxygen, superoxide, hydroxyl, alkyl, and proxy radicals [63].

### Enzymes

**Fig. 7:**
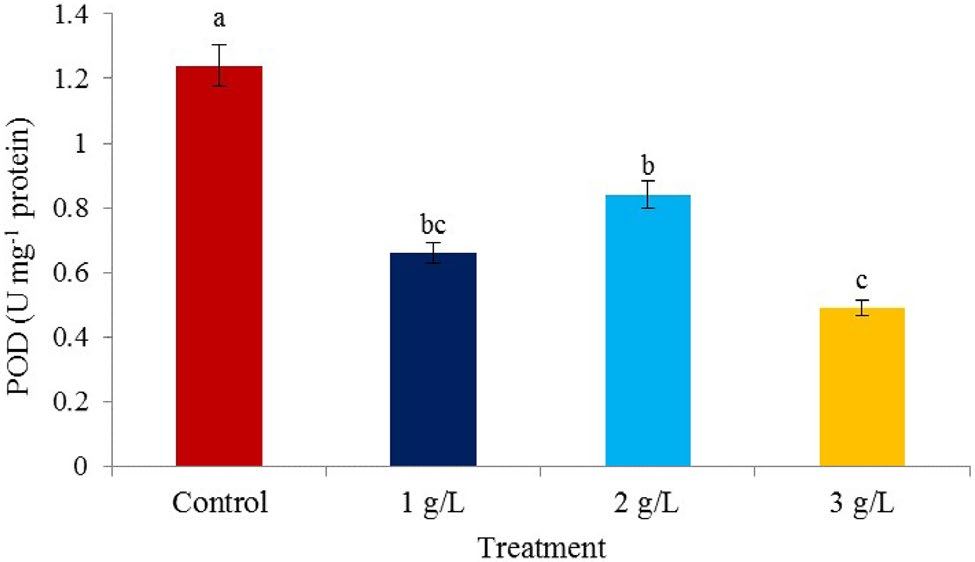
The effect of different *Ascophyllum nodosum* seaweed extract concentrations on peroxidase (POD) enzyme in pistachio “Kaleh-Ghoochi”

The effect of the concentration of seaweed extract consumption on the level of peroxidase enzyme activity was significant, and the time of consumption of seaweed extract and mutual effects did not affect the peroxidase activity (Table 1). The highest peroxidase activity was observed in the control treatment (1.24 µmol APX min^− 1^ mg^− 1^ protein) and the lowest was observed in the 3 g/L seaweed extract treatment (0.49 µmol APX min^− 1^ mg^− 1^ protein) (Fig. 7). Hadavi et al. [64] investigated the enzymatic activity of pistachio kernels only up to the 12th day and concluded that according to the specific enzymatic activity, the kernel is defensive in the first phase and has not yet entered the second phase until the 12th day. This can be justified by the fact that first by increasing the peroxidase enzyme content, the plant strengthens its primary defense barriers, such as the cell wall, and then, by decreasing the enzyme content and increasing the accumulation of its substrate i.e. hydrogen peroxide, it triggers new defense signals. In other words, in the first stage, by increasing the peroxidase enzyme, and in the next stage, by decreasing its content, the plant initiates different defense responses against stresses. The above results can be justified in this way that the treatments of the special enzyme activity plan have been chosen to deal with the stress.

**Fig. 8:**
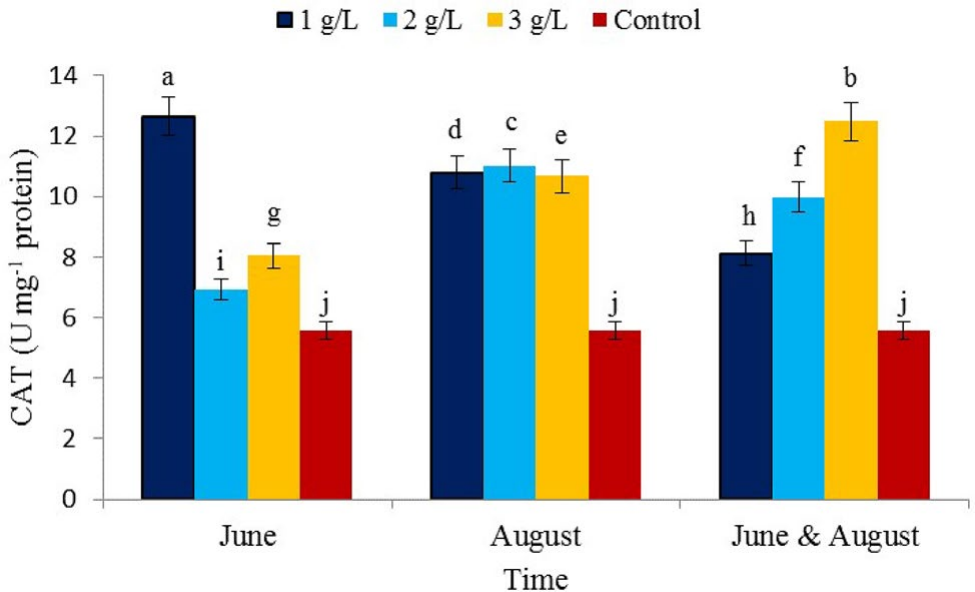
The effect of the time of application of *Ascophyllum nodosum* seaweed extract on catalase (CAT) enzyme in pistachio “Kaleh-Ghoochi”

The effect of the concentration of seaweed extract consumption and the reciprocal effect (concentration × consumption time) on the level of catalase enzyme activity was significant, while the consumption time of seaweed extract did not affect catalase activity (Table 1). The consumption of seaweed extract in the treatment of 1 g/L and the time of consumption in June showed the highest level of catalase activity (12.66 µmol APX min^− 1^ mg^− 1^ protein) and the lowest activity in the control treatment (5.57 µmol APX min^− 1^ mg^− 1^ protein) (Fig. 8). Considering the role of catalase enzyme in increasing stress resistance, it can be concluded that seaweed extract has a positive effect on stress control in pistachio tree. Catalase belongs to the group of oxidoreductase enzymes and iron-containing proteins that can directly convert hydrogen peroxide into water and oxygen and completely remove the toxicity of this oxygen-free radical. *A. nodosum* seaweed extract plays an important role in inhibiting the formation of free radicals in the initiation stage of oxidation and release of the free radical chain reaction as an electron donor [65].

**Fig. 9:**
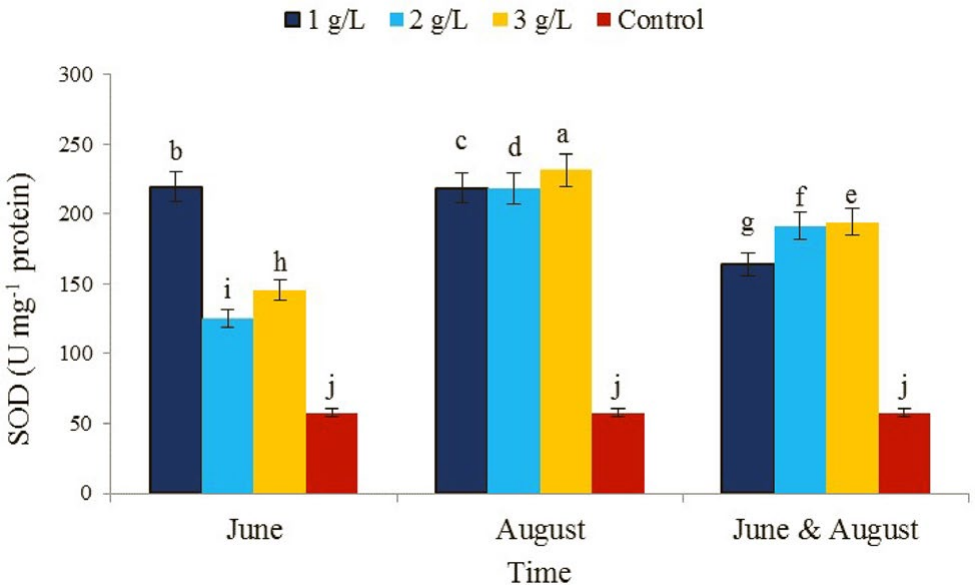
The effect of the time of application of *Ascophyllum nodosum* seaweed extract on superoxide dismutase (SOD) enzyme in pistachio “Kaleh-Ghoochi”

The effect of concentration and time of consumption of seaweed extract alone as well as the interaction effect (concentration × time of consumption) on the level of superoxide dismutase enzyme activity was significant (Table 1). The consumption of seaweed extract in the treatment of 3 g/L and the time of consumption in August showed the highest level of superoxide dismutase activity (231.4 µmol APX min^− 1^ mg^− 1^ protein) and the lowest activity in the control treatment (57.4 µmol APX min^− 1^ mg^− 1^ protein) (Fig. 9). Enzyme antioxidant systems such as superoxide dismutase protect cells against the effects of reactive oxygen species and oxidative stress [66]. There are reports regarding the increase in the activity of superoxide dismutase under the influence of marmarine treatment in stressful conditions [67]. On the other hand, seaweed extract can activate enzymatic and non-enzymatic antioxidant pathways due to its antioxidant properties [68]. Abscisic acid is one of the plant hormones in the structure of seaweed [69]. Studies have shown that abscisic acid is involved in the induction of superoxide dismutase gene expression [70].

**Fig. 10:**
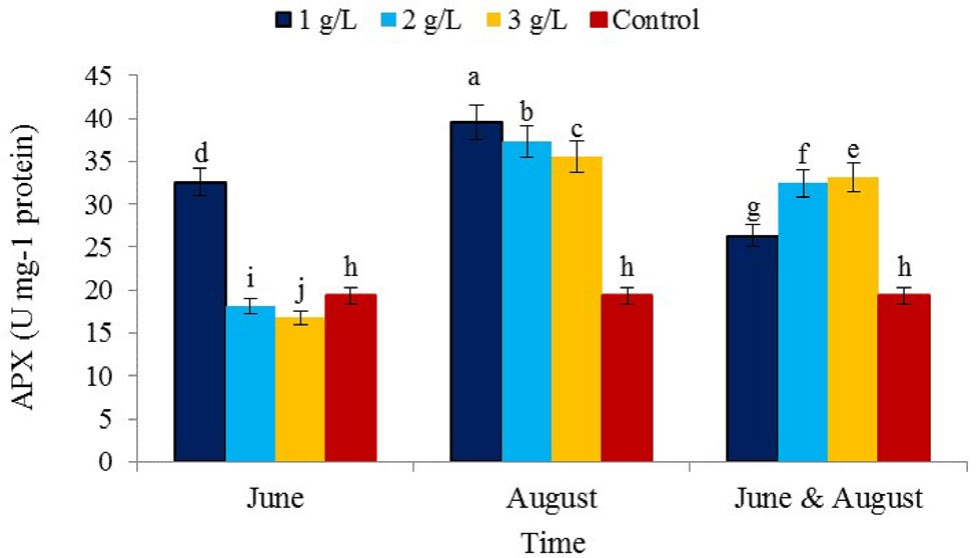
The effect of the time of application of *Ascophyllum nodosum* seaweed extract on ascorbate peroxidase (APX) enzyme in pistachio “Kaleh-Ghoochi”

The effect of consumption concentration and consumption time of seaweed extract as well as the interaction effect (concentration × consumption time) on the level of ascorbate peroxidase enzyme activity was significant (Table 1). The activity of ascorbate peroxidase enzyme increased with the consumption of seaweed extract in the treatment of 1 g/L and the time of consumption in August (39.53 µmol APX min^− 1^ mg^− 1^ protein) and the lowest activity was observed in the treatment of 3 g/L and the time of consumption in June (16.78 µmol APX min^− 1^ mg^− 1^ protein) (Fig. 10). The increase in the activity of the ascorbate peroxidase enzyme in stress conditions is due to the increase in reactive oxygen species, which by activating the message transduction pathways, increases the expression of antioxidant enzymes genes and increases the activity of these enzymes [60]. Seaweed extract significantly increased the activity of superoxide dismutase and ascorbate peroxidase enzymes under stress conditions. The use of seaweed extract increased drought stress tolerance by alleviating the effects of drought stress and increasing antioxidants [71].

## Conclusion

Considering the development of the pistachio tree cultivation in non-native areas and the tree facing biotic and abiotic stresses, investigating the effect of seaweed extract on stress-related traits is of particular importance to increase the tolerance of this crop. According to the results of the present research, foliar spraying with *A. nodosum* seaweed extract and the time of application in different growth stages of pistachio tree showed positive effects on the characters of increasing resistance to ebiotic and abiotic stresses. *A. nodosum* seaweed extract and organic matter played an important role in improving the biochemical traits of pistachio and stress-related traits and caused the improvement of the measured factors compared with the control treatment. In the present research, it was found that the treatment of 1 g/L *A. nodosum* seaweed had a significant effect on carbohydrate, protein, and antioxidant activity, and the interaction effects (concentration × time of use) with the treatment of 3 g/L in August can have a better effect on most traits related to stress tolerance (total phenol, flavonoid, catalase enzymes, superoxide dismutase, and ascorbate peroxidase) in pistachio cultivar “Kaleh-Ghoochi”.

## Data Availability

The data that support the findings of this study are available from the corresponding author upon reasonable request.

## Declarations

### Statement specifying permissions

For this study, we acquired permission to study pistachio (*Pistacia vera* L.) issued by the Agricultural and Natural Resources Ministry of Iran.

### Statement on experimental research and field studies on plants

The either cultivated or wild-growing plants sampled comply with relevant institutional, national, and international guidelines and domestic legislation of Iran.
